# Benchmarking techniques for stereotactic body radiotherapy for early-stage glottic laryngeal cancer: LINAC-based non-coplanar VMAT vs. Cyberknife planning

**DOI:** 10.1186/s13014-019-1404-z

**Published:** 2019-11-04

**Authors:** You Zhang, Tsuicheng Chiu, Jeffrey Dubas, Zhen Tian, Pam Lee, Xuejun Gu, Yulong Yan, David Sher, Robert Timmerman, Bo Zhao

**Affiliations:** 1 Department of Radiation Oncology, UT Southwestern Medical Center, Dallas, TX 75390 USA; 2 Winship Cancer Institute, Emory University, Atlanta, GA 30322 USA

## Abstract

**Introduction:**

Stereotactic body radiation therapy (SBRT) was found effective in treating laryngeal cancer with only five treatment fractions by a recent clinical trial (NCT01984502, ClinicalTrials.gov). Nevertheless, this trial used the Cyberknife system, which is not widely accessible enough to benefit all patients affected by laryngeal cancer. Our study investigates the feasibility of larynx SBRT treatment planning on a conventional gantry-based LINAC and compares its plan quality with that from the Cyberknife.

**Materials & methods:**

Ten larynx SBRT cases were originally treated by Cyberknife using fixed cones in our institution, with plans created and optimized using the Monte-Carlo algorithm in the MultiPlan treatment planning system. These cases were retrospectively planned in the Eclipse planning system for a LINAC with the same prescription dose. We used volumetric modulated arc therapy (VMAT) for larynx SBRT planning in Eclipse and incorporated non-coplanar arcs to approach the Cyberknife’s large solid angle delivery space. We used both anisotropic analytical algorithm (AAA) and Acuros XB (AXB) algorithm for dose calculation and compared their accuracy by measurements on an in-house larynx phantom. We compared the LINAC VMAT plans (VMAT-AAA and VMAT-AXB) with the original Cyberknife plans using dosimetric endpoints such as the conformity index, gradient indices (R50, R20), OAR maximum/mean doses, and the monitor units.

**Results:**

Phantom measurement showed that both the AAA and the AXB algorithms provided adequate dose calculation accuracy (94.7% gamma pass rate on 2%/2 mm criteria for AAA vs. 97.3% for AXB), though AXB provided better accuracy in the air cavity. The LINAC-based VMAT plans achieved similar dosimetric endpoints as the Cyberknife planning, and all plans met the larynx SBRT dosimetric constraints. Cyberknife plans achieved an average conformity index of 1.13, compared to 1.20 of VMAT-AXB and 1.19 of VMAT-AAA. The VMAT plans spared the thyroid gland better with average Dmean of 2.4 Gy (VMAT-AXB) and 2.7 Gy (VMAT-AAA), as compared to 4.3 Gy for Cyberknife plans. The VMAT-AAA plans had a slightly lower contralateral arytenoid Dmax (average: 15.2 Gy) than Cyberknife plans (average: 17.9 Gy) with statistical significance, while the contralateral arytenoid Dmax was similar between VMAT-AXB and Cyberknife plans with no statistically significant difference. Cyberknife plans offered slightly better R50 (average: 5.0) than VMAT-AXB (5.9) and VMAT-AAA (5.7) plans. The VMAT plans substantially reduced the plan MUs to less than 1/3 of the Cyberknife plans, and the differences were statistically significant. The other metrics were similar between VMAT and Cyberknife plans with no statistically significant differences.

**Conclusions:**

Gantry-based LINACs can achieve similar plan quality to Cyberknife systems. Treatment outcome with both methods remains to be investigated.

## Introduction

An estimated 13,000 new cases of laryngeal cancer were diagnosed in the United States in 2016 alone, making it the most common non-cutaneous head and neck malignancy [1]. Most laryngeal cancers occur around the true vocal cord and the glottis larynx region, and most are detected at early stages and can be cured by mono-modality treatments. Radiation therapy, as a local therapy technique, has been highly effective in treating early-stage laryngeal cancer, with local control rates around 90% for Tis and T1 stage tumors, and over 70% for T2 stage tumors [2]. However, conventional radiation therapy treatments for laryngeal cancers usually use opposed-lateral or wedged-pair beams, which result in considerable high dose irradiation to normal tissues. Population-based studies have suggested an increase in late risk of ischemic events following radiation therapy of head and neck cancers [3, 4], presumably from high dose irradiation of carotid arteries. Radiation therapy as a treatment for laryngeal cancer is also facing competition from other surgical alternatives, such as trans-oral laser excision, which removes the gross disease and often preserves adjacent portions of the laryngeal skeleton and mucosa. In addition, current larynx radiation therapy normally requires a long treatment course of 30–33 fractions spanning 5–6 weeks, which can be costly and inconvenient. A hypofractionation scheme would reduce the number of fractions to lower the treatment cost and improve patient convenience. Some previous studies have investigated the feasibility of reducing the total number of fractions of larynx radiotherapy to 25–28, and they have achieved equivalent or even better local control rates without increasing toxicity [5, 6]. These promising results suggest further hypofractionation may achieve better local control and help to minimize the treatment length and cost.

Stereotactic body radiation therapy (SBRT) requires substantially fewer treatment fractions than conventional radiotherapy. Using advanced image guidance and motion control for margin reduction, SBRT may also lower the dose to nearby organs-at-risk (OARs) while simultaneously increasing dose potency to tumors. Thus, SBRT presents a possible solution to the challenges of improving laryngeal cancer treatment with radiation therapy. Our institution recently performed a phase I study of larynx SBRT using the Cyberknife system (Accuray, Sunnyvale, CA) [7, 8]. The study evaluated the feasibility of using SBRT to treat only the involved site of disease plus a 3 mm margin by large-dose, highly-focal radiation fields. The phase I trial has yielded very encouraging results, with local control rates as good as conventional therapy (> = 80%) [8]. We chose the robotic Cyberknife system [9] to deliver radiotherapy by considering several advantages, such as near real-time target tracking capability (offered by the orthogonal x-ray imaging system) and improved dose conformity from non-coplanar beam delivery [10].

Motivated by the encouraging results of the phase I trial, we recently initiated a phase II trial to further evaluate the efficacy of the larynx SBRT technique. Before conducting this trial, we explored the potential of using a gantry-based LINAC systems as an alternative treatment platform for larynx SBRT. Such LINACs are more widely accessible and can benefit a much larger patient population than the Cyberknife. LINAC can provide 3D visualization of soft tissues from CBCT imaging for localization and set up. Some LINACs also have near real-time imaging/localization capacity via technologies like MV cine imaging, ExacTrac [11] (BrainLAB AG, Heimstetten, Germany), AlignRT [12] (Vision RT Ltd., London, UK), Calypso [13] (Varian Medical Systems, Palo Alto, CA), MR-LINAC [14] and etc. All these attributes make a LINAC a potential alternative platform for the phase II study.

In this study, we investigated the feasibility and quality of larynx SBRT planning on a conventional gantry-based LINAC. We proposed to use non-coplanar volumetric-modulated arc therapy (VMAT) beams for LINAC treatment planning. Volumetric-modulated arcs [15] enable more angular coverage than intensity-modulated static beams to preserve planning target volume (PTV) coverage while depositing the dose more uniformly across the whole volume. Using non-coplanar arcs allows more degrees of freedom to approach a large solid angle treatment similar to Cyberknife [10]. VMAT also provides relatively fast delivery to potentially reduce the effect of intra-fractional target motion on delivered doses. Ten patients with laryngeal cancer treated in our Cyberknife SBRT phase I trial were retrospectively replanned in the Eclipse treatment planning system (TPS) (Varian Medical Systems, Palo Alto, CA) for static dosimetry comparison [16]. For Cyberknife planning, we used the Monte-Carlo algorithm for dose calculation because of the air cavity within and/or around the treatment volume. For LINAC planning in Eclipse, we used the anisotropic analytical algorithm (AAA [17]), as it is the most widely used. However, for fair comparison with the Cyberknife plans, we also used the Acuros XB (AXB) algorithm for LINAC plan optimization and dose calculation, because it agrees better with Monte-Carlo than AAA [18, 19]. We also performed an end-to-end phantom test to validate the accuracy of AXB and AAA for larynx dose calculation. We compared the Cyberknife and LINAC VMAT plans using dosimetric endpoints such as the conformity index (CI) [20], OAR maximum/mean doses, R50 (ratio of the 50% isodose volume of the prescription dose to the PTV), R20 (ratio of the 20% isodose volume of the prescription dose to the PTV), and the monitor units (MU).

## Materials and methods

### Validating the dose calculation engine for eclipse: AXB vs. AAA

The larynx contains air pathways, so the PTV for larynx SBRT usually includes an air cavity. For accurate dose calculation and plan evaluation in these inhomogeneous areas, we used the Monte-Carlo algorithm to calculate the dose maps for Cyberknife plans. However, Monte-Carlo dose calculation is not an option in the Eclipse TPS for photon beams. The most widely used algorithm in Eclipse, AAA, has been found to be less accurate in inhomogeneous regions, especially at the air-tissue interface, than in homogeneous regions [19]. The AXB algorithm is a dose calculation algorithm offered in Eclipse as a Monte-Carlo alternative. AXB uses the linear Boltzmann transport equation to solve the dose distribution map. It has been theoretically proven that the linear Boltzmann technique would converge to the same solution as the Monte-Carlo algorithm if infinitely small grids were used [21]. We compared the dose calculation accuracy of AXB and AAA through dosimetric measurements on an anthropomorphic larynx head and neck phantom with air pathways to determine which algorithm is more accurate and whether either (or both) is accurate enough to use with the phase II larynx protocol patients. This larynx phantom was manufactured based on the CT image of a real patient with contours mapped. In detail, the body contours and the larynx air cavity contours on each CT slice were printed together onto papers with the same scale. The papers were then overlaid onto wax slabs of the same thickness as the CT slice (one-by-one), to carve out the body and the air cavity on each wax slab. After all the slabs were carved, they were stacked together to make a volumetric phantom. A larynx SBRT plan was generated for the phantom as described below using the AAA algorithm for plan optimization and dose calculation. The plan was re-calculated using the AXB algorithm. We inserted a radiochromic EBT3 film (Ashland Advanced Materials, Bridgewater, NJ) at the level of the PTV around narrow air tissue interface to measure the actual delivered dose. The film batch was carefully calibrated for absolute dosimetry. Before beam delivery, a CBCT was acquired to determine the actual location of the film for dose comparison. The measured dose was compared with the doses calculated by AXB and AAA through gamma analysis [22].

### Planning study

#### Patient selection, target definition, and prescription

We retrospectively studied ten patients diagnosed with cTis-T2N0M0 stage glottic larynx carcinoma, under a departmental umbrella protocol approved by the internal review board (IRB). These patients were treated in our Phase 1 Cyberknife trial (NCT01984502, ClinicalTrials.gov). The gross tumor volume (GTV) was contoured on high resolution free-breathing simulation CT scans. From the 4D-CT acquired at the time of simulation, an internal target volume (ITV) was constructed to include the GTV plus the motion observed from the 4D-CT. The clinical target volume (CTV) included the ITV plus a 2 mm geometric expansion. The CTV might also include the ipsilateral arytenoid, the ipsilateral/bilateral vocal cord(s), the anterior commissure, and the ipsilateral/bilateral paraglottic space(s), depending on the relative distances and the stage/extension of the disease. The PTV was formed by expanding the CTV by a 3 mm uniform margin in all directions. All treatment plans were developed and treatments were performed on the Cyberknife system based on the MultiPlan TPS (Accuray, Sunnyvale, CA) with a total prescribed dose of 42.5 Gy delivered in 5 fractions.

#### Planning objectives and techniques

For both Cyberknife and VMAT planning, the PTV should be covered by at least 95%, but not more than 120%, of the prescription dose in order to maintain homogeneity. The following critical structures were contoured as OARs: left/right carotid artery, contralateral arytenoid, thyroid gland, spinal cord, and skin (5 mm inner-ring from external body contours, excluding PTV). The detailed constraints and objectives for the PTV and OARs are shown in Table 1.

**Table 1:** Planning constraints/objectives for the PTV and OARs. The maximum dose is the point maximum dose defined to 0.035 cc

Structure/Organ	Evaluation	Goal	Unit
PTV	V_Rx_	>= 95%	V%
	D_max_	<= 48.9	Gy
	D_max_	<= 51.0 (required)	Gy
	Conformity index	<= 1.3 (required)	N.A.
Left/Right Carotid Artery	D_max_	<= 23.0	Gy
	D_mean_	<= 11.2	Gy
Spinal Cord	D_max_	<= 20.0 (required)	Gy
Skin	D_max_	<= 42.5	Gy
Contralateral Arytenoid	D_max_	<= 23.0	Gy
Thyroid Gland	D_mean_	<= 15.3	Gy

All the doses shown here are recommended objectives (not required), except where specifically noted

#### Cyberknife planning

The ten larynx SBRT patients were originally treated using a Cyberknife robotic SBRT platform. Because of the small PTV sizes (range: 2.7 cc – 11.1 cc, median: 4 cc), the Cyberknife plans used fixed cones [23] instead of the iris cones or the multileaf collimators (MLCs). The fixed cones are preferred over the iris cones for small targets in need of cone sizes smaller than 10 mm [24]. Sizes of the used fixed cones in our Cyberknife larynx SBRT plans were 5 mm, 7.5 mm or a combination of the two. Moreover, fixed cones may provide better target conformity indices as compared to the MLCs according to a published study [25]. The treatment plans were created and optimized using the Monte-Carlo algorithm [26] in the MultiPlan TPS using the sequential optimization mode. All Cyberknife plans were approved by the attending physician, and treatments were successfully delivered.

#### LINAC planning

**Fig. 1:**
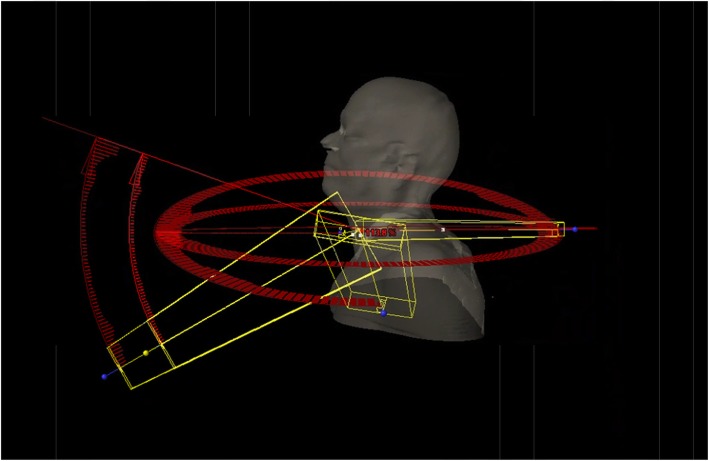
Image rendering of the 5-arc template for LINAC larynx SBRT planning

We exported the images and structure sets of the larynx patients from the MultiPlan TPS to the Eclipse TPS and used Eclipse for larynx SBRT planning on a LINAC with 6-degree couch rotation capability (TrueBeam, Varian Medical Systems, Palo Alto, CA). The LINAC was equipped with a Millennium 120 leaf MLC, featuring a central resolution of 5 mm per leaf by 40 leaf pairs and a peripheral resolution of 10 mm per leaf by 20 leaf pairs, for a maximum field size of 400 mm by 400 mm. To maximize PTV coverage and OAR avoidance, we introduced couch kicks into the plan to generate non-coplanar partial arcs to approach a large solid angle beam entry geometry. The span of each arc was optimized to avoid potential collisions and to avoid directly irradiating anatomies such as the chins during delivery. After multiple trial-and-error runs, we finalized a 5-arc template that considered both plan quality and delivery efficiency (Table 2, Fig. 1).

**Table 2:** The 5-arc plan template for LINAC larynx SBRT planning

Field	Energy	Gantry Rotation	Collimator Angle	Couch Angle
Arc 1	6x	270° CW 179°	10°	10°
Arc 2	6x	90° CCW 181°	350°	350°
Arc 3	6x	330° CW 20°	10°	90°
Arc 4	6x	330° CW 20°	10°	90°
Arc 5	6x	179° CCW 181°	350°	0°

#### Evaluation

We retrospectively designed non-coplanar VMAT plans in Eclipse using both AXB (VMAT-AXB) and AAA (VMAT-AAA) algorithms for all the larynx patients. We set the dose grid size of the VMAT plans to 1 mm to match that of the original Cyberknife plans and to accommodate the small PTV. The AXB algorithm calculated and reported the dose to medium to match the Monte-Carlo algorithm employed by the Cyberknife. To enable direct comparison, we normalized both VMAT and Cyberknife plans to the same PTV coverage level, at which 95% of the PTV volume received 100% of the prescription dose. We compared the plans using metrics including the maximum PTV dose, maximum or mean doses to various OARs (left/right carotid artery, spinal cord, skin, thyroid gland, and contralateral arytenoid), R50, R20, conformity index, and total plan MUs. For plan comparison, we exported all MultiPlan dose maps to Eclipse and extracted the dose metrics of both plans from Eclipse to minimize potential variations caused by different interpolations of volume/dose between Eclipse and MultiPlan.

We compared the VMAT-AXB plans and VMAT-AAA plans to the original Cyberknife plans. Statistical significance was assessed by the Wilcoxon signed-rank test with the statistical significance level defined at *p* < 0.025, to which Bonferroni correction was applied to account for multiple (2) comparisons.

## Results

### Phantom dosimetric study

**Fig. 2:**
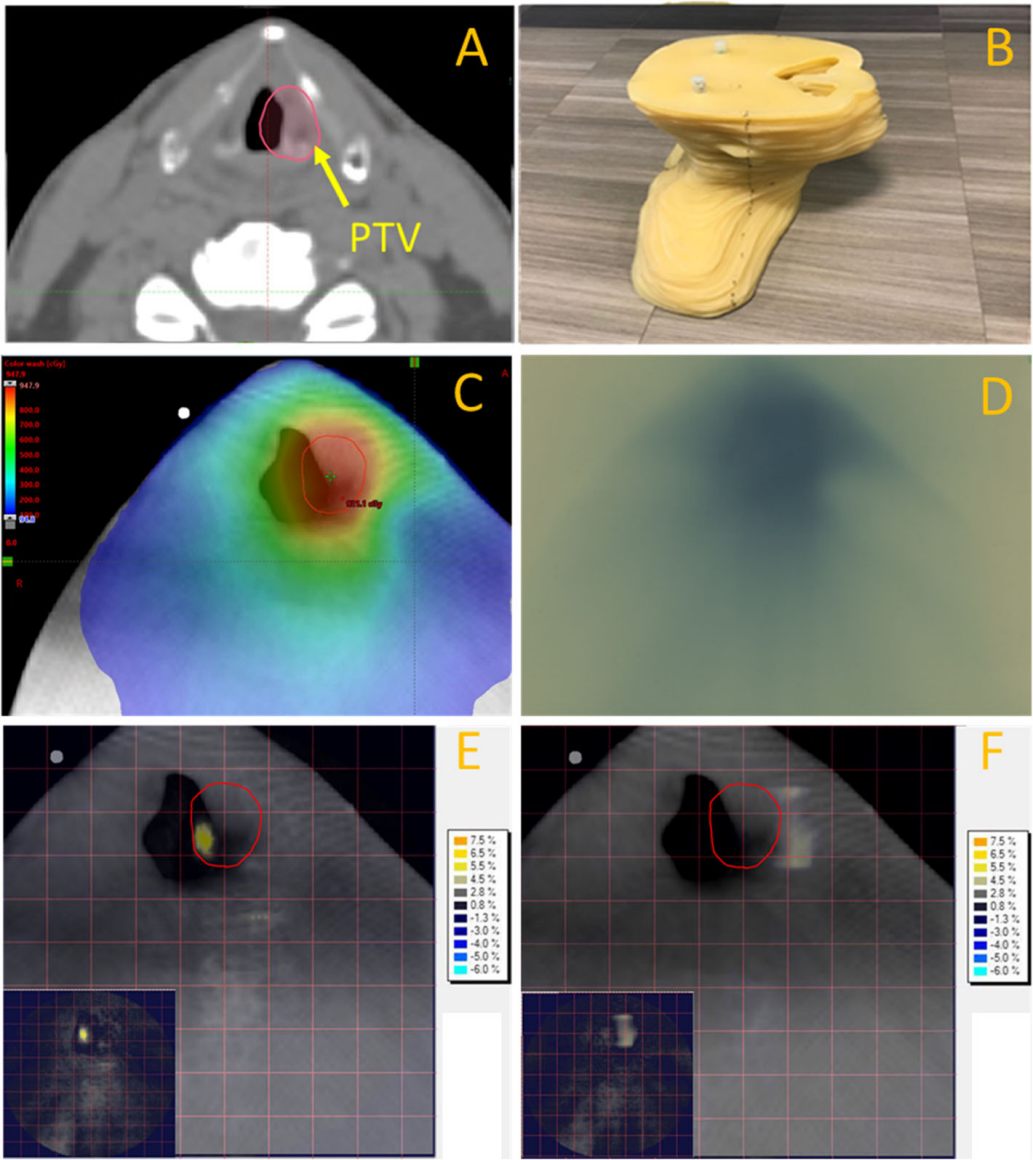
(**a**) CT slice of an example larynx SBRT patient, where the PTV contour covers a considerable amount of air cavity. (**b**) Our in-house larynx wax phantom used for dose measurement and dose accuracy validation/comparison. (**c**) Mapping of a larynx SBRT plan onto the larynx phantom and the corresponding dose distribution calculated by AXB. (**d**) Dose distribution measured by the EBT3 film. The film was sandwiched between the phantom slabs at the slice location of (**c**). (**e**) 2D dose difference map for the Eclipse AAA algorithm. The brighter pixels indicate larger dose differences, as shown by the color bar. (**f**) 2D dose difference map for the Eclipse AXB algorithm. The PTV contour was overlaid onto both (**e**) and (**f**). The difference maps (without underlying CTs) were attached at the lower-left corners of (**e**) and (**f**)

The in-house phantom study comparing the AXB and AAA algorithms demonstrated that both provided adequate dose calculation accuracy (Fig. 2). With the conventional criterion of 3%/3 mm [22], the gamma pass rates based on the absolute dose comparison were 100% for AXB and 99.7% for AAA. Under a stricter gamma criterion (2%/2 mm), our analysis still yielded over 97.3% pass rate for AXB and 94.7% for AAA. For both 3%/3 mm and 2%/2 mm criteria, the dose differences were normalized to the global dose maximum. In addition, a 10% low dose threshold was applied for all evaluations to exclude the very low dose points. The AXB algorithm calculated doses more accurately than AAA for the air cavity region, which agrees with previous studies [19]. The accuracy of the Cyberknife-calculated dose distributions via the Monte-Carlo algorithm has been verified in a separate study [7].

### Cyberknife and LINAC-VMAT plan comparison

**Fig. 3:**
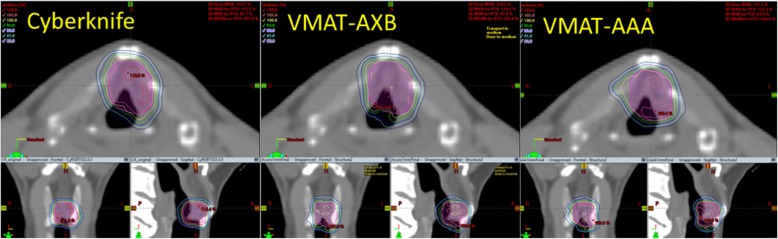
Isodose map comparisons in three views between an original Cyberknife plan and the non-coplanar VMAT LINAC plans (VMAT-AXB and VMAT-AAA) for one studied case

**Fig. 4:**
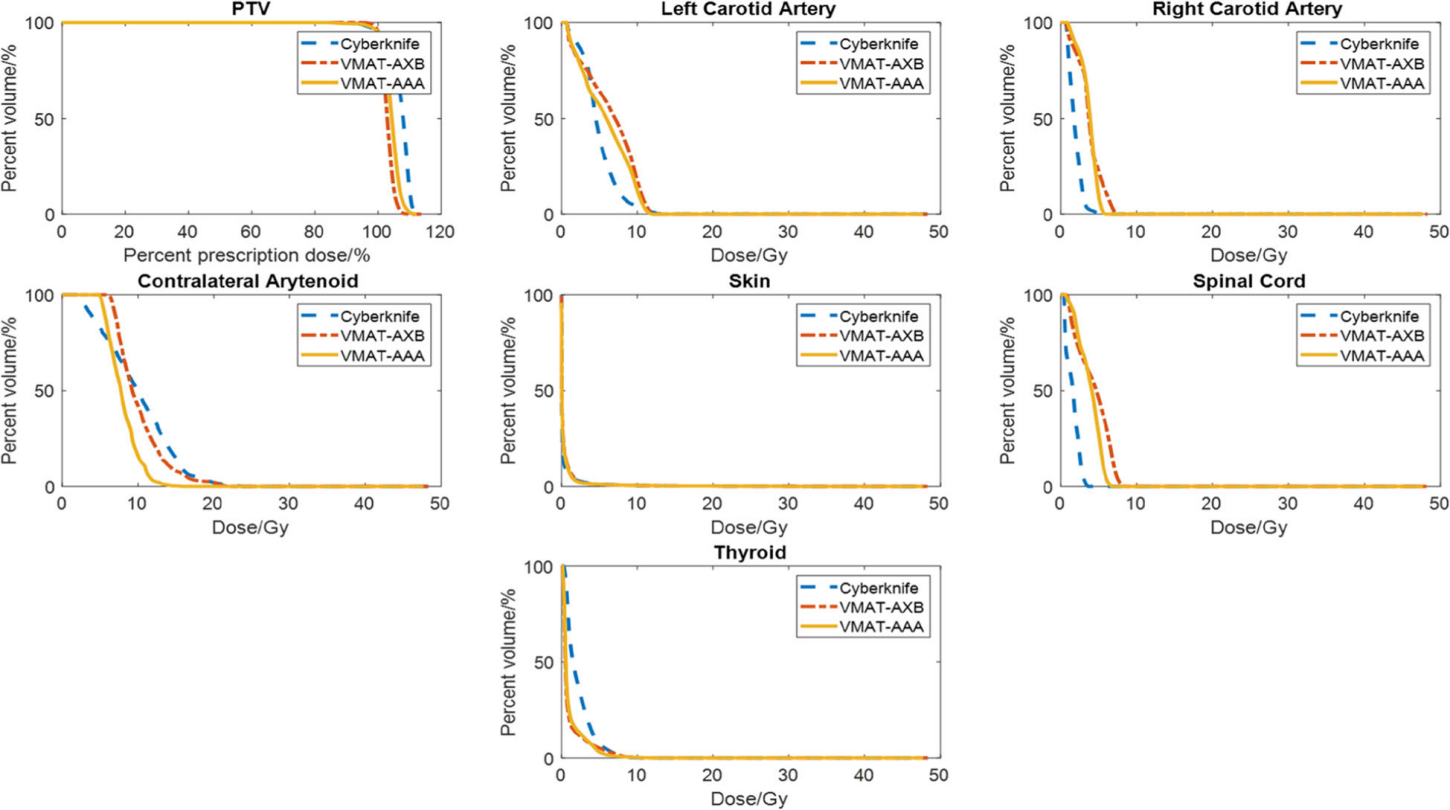
DVH comparison between an original Cyberknife plan and the non-coplanar VMAT LINAC plans (VMAT-AXB and VMAT-AAA) for one studied case

We compared the isodose maps of non-coplanar VMAT plan dose distributions with Cyberknife dose distributions and presented one case, shown in Fig. 3. All three plans delivered highly conformal dose distributions to the target. The dose distribution differences between AAA and AXB plans were caused by the differences of optimization paths and the dose calculation engine differences. The LINAC plans achieved slightly less dose spillage in the anterior-posterior position than the Cyberknife plan. However, the sagittal and coronal views revealed that the dose spillage in the superior-inferior direction was more pronounced for the LINAC plans. In general, the dose distributions were visually similar between Cyberknife and LINAC plans, demonstrating that all can achieve tight target coverage. For the same patient, we also plotted the DVH curves of the PTV and OARs for comparison (Fig. 4). These curves show that the VMAT plans (VMAT-AXB and VMAT-AAA) achieved the same PTV coverage as the original Cyberknife plan (95% target covered by 100% prescription dose), while the PTV of the Cyberknife plan was generally hotter. The OAR sparings were similar between the VMAT plans and the Cyberknife plan: the VMAT plans spared the thyroid better, while the Cyberknife plan spared the right carotid artery and spinal cord better.

**Fig. 5:**
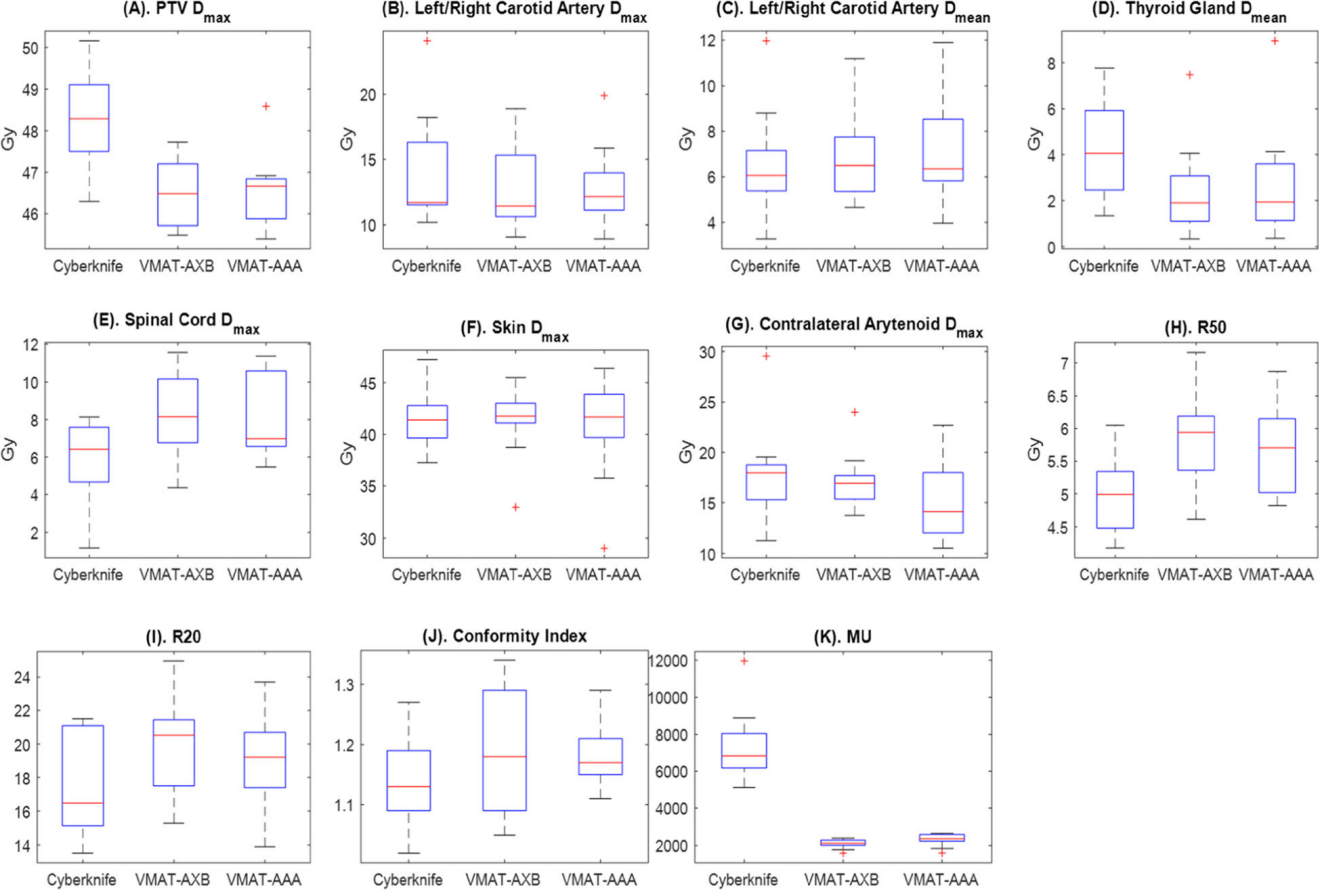
Distribution boxplots of all metrics evaluated in the plan comparison study for the Cyberknife, VMAT-AXB, and VMAT-AAA plans. Each boxplot contains 10 data points. In each boxplot, the upper edge, central line, and lower edge of the box represent the 75th percentile (Q3), median, and 25th percentile (Q1) of the data, respectively. The lower whisker extends to the datum no smaller than Q1 − 1.5 × (Q3 − Q1), and the upper whisker extends to the datum no larger than Q3 + 1.5 × (Q3 − Q1). The “+” in the plots are outliers outside the whiskers

The plots of all metrics in Fig. 5 revealed the similarity of dosimetric outcomes between the different planning methods. All planning techniques met the constraints required in Table 1. The maximum PTV dose was higher for Cyberknife, than for VMAT-AXB (*P* < 0.01) and VMAT-AAA (P < 0.01) (Table 3). Four of the Cyberknife plans had PTV maximum doses higher than 48.9 Gy, the recommended limit shown in Table 1. However, all were within the mandatory limit of 51.0 Gy (Table 1) and were approved by the physicians for treatment. All VMAT-AXB/VMAT-AAA plans had PTV maximum doses within the recommended limit of 48.9 Gy. The average mean thyroid dose of Cyberknife plans was also slightly higher due to its anterior beam pathway, than those of the VMAT-AXB (*P* < 0.002) plans and the VMAT-AAA (*P* < 0.02) plans (Table 3), but all were well below the limit of 15.3 Gy (Table 1). Both the Cyberknife and the VMAT-AXB/VMAT-AAA plans had spinal cord doses well below the 20 Gy constraint, probably because of the anterior location of the PTV. Because there were no posterior beams, the Cyberknife plans had lower spinal cord doses on average (Fig. 5), but these differences were not statistically significant (*P* > 0.025 for Cyberknife vs. VMAT-AXB and P > 0.025 for Cyberknife vs. VMAT-AAA, Table 3). The maximum dose to the contralateral arytenoid was lower for VMAT-AAA than for Cyberknife, with statistical significance (*P* < 0.02, Table 3), while no statistically significant difference was found between the VMAT-AXB and Cyberknife plans. It should be noted, however, that the AAA algorithm may not accurately calculate the actual dose to be delivered. The Cyberknife plans were slightly better on the R50 metric, with statistical significance, than the VMAT-AXB (*P* < 0.002) and VMAT-AAA (*P* < 0.01) plans (Table 3), indicating that the dose distributions of the Cyberknife may be more compact with sharper dose fall-offs because we used fixed cones. Nonetheless, the differences between plans were small and not statistically significant for the R20 metric and the conformity index. The MUs for both the VMAT-AXB and VMAT-AAA plans were substantially lower than (less than 1/3 of) the Cyberknife plans, with statistical significance (P < 0.002 for Cyberknife vs. VMAT-AXB and P < 0.002 for Cyberknife vs. VMAT-AAA, Table 3). In general, the dosimetric results were similar between the Cyberknife plans and the non-coplanar VMAT plans, which demonstrates the feasibility of treating larynx SBRT patients using conventional gantry-based LINACs to achieve similar plan quality. The dosimetric metrics were mostly similar between VMAT-AXB and VMAT-AAA plans. Overall, the results showed that VMAT-AXB achieved plan quality similar to VMAT-AAA after optimization, even though the air cavity posed additional challenges for PTV coverage and OAR sparing.

**Table 3:** Dosimetric endpoint comparison between Cyberknife and VMAT plans

Average (S.D.)	PTV D_max_ (Gy)	Left/Right Carotid Artery D_max_ (Gy)	Left/Right Carotid Artery D_mean_ (Gy)	Thyroid Gland D_mean_ (Gy)	Spinal Cord D_max_ (Gy)	Skin D_max_ (Gy)
Cyberknife	48.3 (1.2)	13.9 (4.4)	6.6 (2.4)	4.3 (2.2)	5.8 (2.2)	41.4 (2.9)
VMAT-AXB	46.5* (0.8)	12.7 (3.1)	7.1 (2.4)	2.4* (2.1)	8.2 (2.2)	41.3 (3.5)
VMAT-AAA	46.6* (0.9)	12.9 (3.1)	7.1 (2.4)	2.7* (2.5)	7.9 (2.2)	40.1 (5.0)
Average (S.D.)	Contralateral Arytenoid D_max_ (Gy)	R50	R20	Conformity index	MU	
Cyberknife	17.9 (4.8)	5.0 (0.6)	17.4 (3.0)	1.13 (0.07)	7333 (1934)	
VMAT-AXB	17.2 (2.9)	5.9* (0.8)	20.2 (3.2)	1.20 (0.10)	2095* (259)	
VMAT-AAA	15.2* (4.0)	5.7* (0.7)	19.2 (3.1)	1.19 (0.05)	2311* (343)	

The metric values were presented as the average of all ten patients studied, with numbers in brackets showing the standard deviations. The “*” mark was added when the difference between the Cyberknife and the VMAT-AXB or between the Cyberknife and the VMAT-AAA was statistically significant (*p* < 0.025)

## Discussion

Previous studies have found that the AXB algorithm calculates doses at inhomogeneous regions more accurately than the AAA algorithm [18, 19]. Our findings in the end-to-end phantom measurement study agreed with those studies. Good overall accuracy was observed for either algorithms on a static phantom (Fig. 2), however AXB provided better dose accuracy in the air cavity region. For larynx PTV, the air cavity is included to compensate motion but not for actual dose disposition. We believe that actual dose deposition in soft tissues will be adequate for either AAA or AXB (Fig. 2) with motion. Since AAA reports dose to water [27], and most clinical outcome studies are based on dose to water [28, 29], our clinicians are more comfortable interpreting the treatment outcome based on AAA dose calculation. We will continue to use AAA as the dose calculation engine for our phase II trial until we gain more experience with AXB. In the future AXB will likely replace AAA as the main dose calculation engine due to its better accuracy.

Our study shows that larynx SBRT may be planned either on Cyberknife or on conventional LINACs with the same target coverage and similar OAR avoidance (Figs. 3, 4 and 5 and Table 3). There are no statistical differences for most evaluation metrics. For metrics where statistically significant differences were identified, VMAT-AXB and VMAT-AAA plans were either slightly better on selected metrics (i.e., maximum PTV dose, mean thyroid gland dose, maximum contralateral arytenoid dose, and MU), or slightly worse (i.e., R50) than the Cyberknife plans. In contrast, a previous study found worse OAR sparing by LINAC-based coplanar IMRT plans than Cyberknife plans [7], likely due to the beam arrangement. In our study, the good LINAC plan quality indicates that using VMAT can evenly distribute the beams to achieve better target coverage, and using non-coplanar geometry can further spare OARs. It is worth to mention that the Cyberknife plans offered more compact dose distributions and better conformity indices by using fixed cones [23]. In contrast, our LINAC used a standard MLC of 5 mm resolution at the isocenter level. A high-definition MLC with a finer leaf width (2.5 mm) may improve the dose fall-off to achieve more compact doses [30] for our proposed non-coplanar VMAT plans. In general, this comparison study suggested that similar plan quality can be achieved on either LINACs or Cyberknife platforms for larynx SBRT treatments, meeting all the dosimetric constraints on Table 1.

It is found that VMAT plans used less than 1/3 of the total MUs of the Cyberknife plans (Table 3). The large MUs associated with Cyberknife plans are partially due to the small cones used in this study, as MUs and treatment time generally decrease with increasing cone size. However, due to the limited size of the larynx PTV, we found using a small fixed cone necessary to maintain the plan quality, especially on the dose conformity index. Although the MUs can be partially correlated with the net beam-on time, they cannot fully represent the overall treatment delivery time, especially when comparing between two different modalities. The treatment delivery time comparison between LINAC and Cyberknife can be complex, depending on many factors including dose rate, intra-fractional imaging and setup correction. It should be acknowledged that the treatment time, especially for LINAC delivery, can vary among institutions with different intra-fractional verification protocols and technology. Different institutions should compare the treatment delivery time between LINAC and Cyberknife based on their own protocols. In general, shorter treatment time potentially reduces the effect of intra-treatment tumor motion or baseline drift [31, 32], though Cyberknife systems could be less susceptible to intra-treatment tumor motion from longer treatment time as compared to LINACs, due to its real-time motion compensation strategy. However, it should also be emphasized that shorter treatment time may also indicate higher dose rate, and higher dose rate may potentially increase the incidence of normal tissue toxicity for larynx SBRT from a radiobiological point of view [33]. Thus careful consideration is warranted in selecting a technology for potential larynx SBRT treatments.

The InCise MLC on the Cyberknife systems may deliver plans more efficiently and help reduce the beam-on time [25]. Our fixed cone plans were all designed and delivered before our clinic adopted and commissioned an InCise MLC for Cyberknife. Retrospectively, we also performed a preliminary study investigating the potential of using the InCise MLC to plan the CK larynx SBRT cases. For the Cyberknife MLC plans, we used the Cyberknife Precision TPS with the latest VOLO optimizer. Similarly, the Monte-Carlo engine was employed for dose calculation. From the study, we found the MLC plans could not meet all objectives/constraints especially the conformity index constraint. For three evaluated patient cases, the MLC plans yielded conformity index all > 1.4, exceeding our protocol’s hard constraint (1.3). Thus we did not further pursue the use of the Cyberknife MLC to generate the larynx SBRT plans for comparison. Such a discrepancy for MLC plans could be caused by several factors: 1) the small PTV size of larynx plans (2.7 cc – 11.1 cc for this study) for which MLC has found challenging in achieving a good quality plan especially on conformity index [25]; 2) the “fluence-to-leaf sequence” optimization strategy for MLC, of which the post-optimization leaf sequencing may lead to inferior plan quality; 3) Cyberknife larynx plans have to employ the Monte Carlo dose calculation algorithm due to the air cavity presence. However, the current VOLO optimizer only applies the Monte-Carlo algorithm in the later stages of MLC plan optimization that include segment weighting adjustment and final dose calculation. Instead, it uses pencil-beam-based algorithms for fluence optimization and leaf adaptation, which may lead to the sub-optimal plans after final Monte-Carlo dose calculation of the MLC plans. In contrast, fixed cone plans do not require fluence optimization. And the optimization of fixed cone plans is driven by Monte Carlo dose calculation. As a result, the final dose of fixed cone plans is close to that achieved during optimization. Nonetheless, these limitations on MLC optimization may be overcome with future algorithm updates, and the potential of Cyberknife MLC plans should be re-assessed in the future.

In this comparison study, we used the same margin recipe for both the LINAC VMAT and the Cyberknife plans. In our phase II larynx SBRT trial (NCT03548285, ClinicalTrials.Gov), we are investigating the use of surface imaging-based motion management strategy for LINAC treatments [36]. The skin surface around the larynx region is tracked as a surrogate of intra-fractional tumor motion. Beam-hold is enabled when the surface motion goes beyond a clinically-defined threshold. This strategy allows continuous motion monitoring in real time, potentially achieving similar motion control capability as Cyberknife and hence a similar margin recipe. Therefore, the dose results reported in this study were based on our clinically-realistic plans, and reflected our clinical practices. However, it should be noted that the margin recipes for LINACs or Cyberknife machines might vary among institutions, which are affected by individual technology capability and institutional policy. One may have to weigh in the potential margin differences when generating the LINAC and CK plans for dose comparison, to determine the most appropriate technology to use.

There are some limitations of our study. This planning study is based on static dose calculation which does not account for motion. There are potentially complex interplay effects between the larynx motion, the air cavity and the small, intensity-modulated treatment fields, which may lead to dose deviations from planning. Measurement studies using a motion phantom are warranted to evaluate the effects of larynx motion, and further compare the gantry-based LINAC with Cyberknife for larynx SBRT treatments. Furthermore, the actual delivered dose cannot be easily tracked on patients. In addition to motion-induced dosimetric uncertainty, the differences in dose calculation engines may also introduce dosimetric uncertainties (for instance, AAA vs. AXB), which is difficult to quantify in a patient-specific or organ-specific manner. A further comparison will rely on a future treatment outcome study.

## Conclusions

In this study, we evaluated the dosimetric quality of LINAC VMAT plans against the Cyberknife plans for early-stage larynx SBRT treatments. In our clinical practice, the same margin recipe was applied for both LINAC and Cyberknife planning. Likewise, same dosimetric objectives and constraints were employed during optimizing with the two planning engines (Eclipse and Multiplan). For VMAT plans, we used both AAA and AXB dose engines for dose calculation and plan optimization, and compared the results. Phantom measurements were performed to further evaluate the accuracy of dose calculations by AAA and AXB. It was found that both AAA and AXB provided adequate overall dose calculation accuracy although AXB was more accurate at the air cavity region. This planning study revealed that a gantry-based LINAC, either with AAA or AXB, can achieve similar dosimetric endpoints as Cyberknife(Monte Carlo dose calculation), by employing non-coplanar VMAT arcs. Future study will evaluate the clinical outcomes of patients treated on these two platforms.

## Data Availability

Please contact the corresponding author for data requests.
